# Holistic multi-class classification & grading of diabetic foot ulcerations from plantar thermal images using deep learning

**DOI:** 10.1007/s13755-022-00194-8

**Published:** 2022-08-26

**Authors:** Shishir Muralidhara, Adriano Lucieri, Andreas Dengel, Sheraz Ahmed

**Affiliations:** 1 Smart Data & Knowledge Services (SDS), German Research Center for Artificial Intelligence (DFKI) GmbH, Trippstadter Strasse 122, 67663 Kaiserslautern, Rhineland-Palatinate Germany; 2 Computer Science Department, TU Kaiserslautern, Erwin-Schroedinger-Strasse 52, 67663 Kaiserslautern, Rhineland-Palatinate Germany

**Keywords:** Diabetis mellitus, Diabetic foot ulceration, Thermography, Deep learning, Image processing, Medical image analysis

## Abstract

**Purpose:**

Diabetic foot is a common complication associated with diabetes mellitus (DM) leading to ulcerations in the feet. Due to diabetic neuropathy, most patients have reduced sensitivity to pain. As a result, minor injuries go unnoticed and progress into ulcers. The timely detection of potential ulceration points and intervention is crucial in preventing amputation. Changes in plantar temperature are one of the early signs of ulceration. Previous studies have focused on either binary classification or grading of DM severity, but neglect the holistic consideration of the problem. Moreover, multi-class studies exhibit severe performance variations between different classes.

**Methods:**

We propose a new convolutional neural network for discrimination between non-DM and five DM severity grades from plantar thermal images and compare its performance against pre-trained networks such as AlexNet and related works. We address the lack of data and imbalanced class distribution, prevalent in prior work, achieving well-balanced classification performance.

**Results:**

Our proposed model achieved the best performance with a mean accuracy of 0.9827, mean sensitivity of 0.9684 and mean specificity of 0.9892 in combined diabetic foot detection and grading.

**Conclusion:**

To the best of our knowledge, this study sets a new state-of-the-art in plantar foot thermogram detection and grading, while being the first to implement a holistic multi-class classification and grading solution. Reliable automatic thermogram grading is a first step towards the development of smart health devices for DM patients.

## Introduction

Diabetes mellitus (DM), or diabetes, is a condition characterized by excessive blood sugar levels. According to the World Health Organization (WHO), over 422 million people worldwide suffer from diabetes [1]. Diabetes results in several complications such as diabetic retinopathy which damages the retina and may result in blindness, diabetic nephropathy which affects the kidney and diabetic neuropathy which causes nerve damage and results in loss of sensation. Another complication that presents itself in Diabetics is ulceration or destruction of tissues of the foot. Diabetic Foot Ulcers (DFUs) are open sores or lesions that will not heal or that recur over a long period of time. The lifetime occurrence of a foot ulceration in diabetic patients is estimated to be up to 25% [2]. Furthermore, foot ulcers reoccur after healing, with a recurrence incidence of 40% within 1 year, 60% within 3 years, and 65% within 5 years [3]. When accompanied by diabetic neuropathy, the patient does not feel any pain and may not realize the presence of an ulcer. Ulcerations are the precursor leading to amputation in more than 85% of major amputations [4].

Proper management and foot care can help in the prevention of foot ulcers. If the onset or occurrence of ulcerations are identified at an early stage, appropriate preventive measures or interventions can be taken to avoid amputations. Prior to the appearance of any foot ulcer, there is an increase in the local temperature due to an underlying inflammatory process [5]. Studies provide robust evidence that in addition to the standard treatment, thermometry of the feet is effective in reduction of the incidence of new foot ulcers [5]. In [6] three methods for monitoring plantar temperature have been identified, namely infrared thermometers, infrared cameras and liquid crystal thermography (LCT). In case of IR thermometers, the users have to measure and record the temperature manually at pre-identified risk points. The LCT technology requires the patient to place their feet on a LCT indicator plate, which describes the temperature distribution of the foot through the imprint. The imprint remains for a short duration and it is compared with a template. Recently, however, IR thermography has emerged as the preferred mode for measuring the temperature owing to its non-invasive and non-contact method.

Infrared thermography uses thermal cameras to detect heat patterns and blood flow. Temperature differences between the feet may indicate the development of foot ulceration [7]. A case study by Bagavathiappan et al. [8] identified an association between plantar temperature and diabetic neuropathy, and substantiated the efficacy of thermography for diabetic foot diagnosis.

**Fig. 1:**
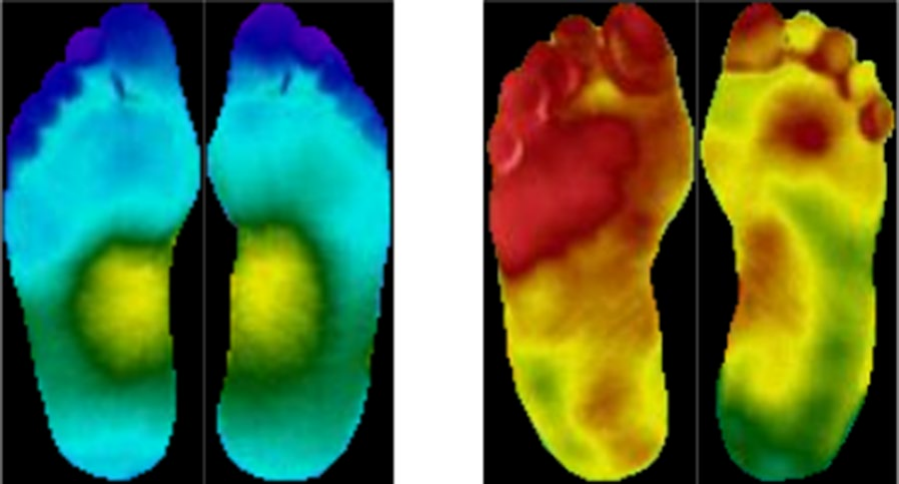
Symmetrical thermal distribution observed in a non-diabetic subject (left) and absent in a diabetic subject (right)

Thermograms reveal the distribution of plantar temperature which is symmetrical in both feet of non-diabetic subjects, with elevated temperature in the arc of the foot, resembling a butterfly pattern. However, in diabetic patients this pattern is not observed. The emphasis is on the temperature distribution across both feet, rather than the observed value of the temperature at a point for determining points of ulceration. Thermograms for a non-diabetic and a diabetic individual are shown in Fig. 1, where the previously discussed thermal distribution patterns can be observed.

There are two popular methods for processing thermograms [9]:

1. Asymmetric analysis
2. Temperature distribution analysis

Asymmetric analysis is based on the butterfly pattern of temperature distribution, where asymmetry in this distribution indicates abnormality. The temperature is measured at a point and compared with the temperature at the same location on the corresponding foot in order to determine a point of ulceration. If the temperature difference is more than 2.2 $^\circ$C, it is registered as a point of ulceration. This method has the disadvantage of not being applicable for patients with deformities or partial foot amputations. The second approach involves analysing the temperature distribution for each foot independently by calculating a reference value with respect to temperature distribution in healthy individuals. It is intrinsically inclusive of patients with deformities or amputation because it does not require comparison between points across the feet.

Several studies [10–13] support the evidence that temperature-monitoring systems constitute feasible and efficient strategies to identify the onset of ulcerations. Deep learning (DL) has shown great success in several medical application domains such as radiology [14], dermatology [15] and opthalmology [16]. A series of works have been published, reporting high performance for classification and grading of diabetes-related diseases such as diabetic retinopathy [17] and diabetic neuropathy [18]. However, the classification and detection of diabetic foot ulcers is still largely underrepresented in the domain of DL-based medical image analysis. To the best of our knowledge, only a single work [19] using DL for DFU classification has been published. There have been several studies using DL, which we have included under related works. Moreover, previous works using ML or DL have largely neglected the consequences of imbalanced data on the classification, most of them considered only limited settings of binary or multi-level classification.

In this work, we follow a new, holistic classification approach considering thermograms from non-diabetic as well as diabetic subjects. This allows our proposed convolutional neural network (CNN)-based system to both distinguish between diabetic and non-diabetic thermograms, based on the deviation from symmetry, while also providing a severity grading of the diabetic thermograms into five distinct classes. In contrast to previous works, we directly address the biased classification that occurs due to the limited availability of mostly imbalanced datasets, and explore the influence of different input encodings. Using five fold cross-validation, we achieve a state-of-the-art performance with a mean accuracy of 0.9827, mean sensitivity of 0.9684 and mean specificity of 0.9892 in combined diabetic foot detection and grading.

The rest of the paper is organised as follows. In “Related works” section, we provide a short review of previous works on the analysis of plantar foot thermograms. “Materials and methods” section briefly describes the dataset used, as well as the classification pipeline of our proposed approach. Experimental investigations along with the results are presented in “Experiments” section. Finally, the results are discussed in “Discussion” section, followed by a conclusion of our work.

## Related works

**Table 1:** Summary of the previous efforts in plantar foot thermogram analysis along with the reported results

Author	Methods	Dataset		Performance metrics				
		D M	C G	Accuracy	Specificity	Sensitivity	Precision	F-Score
Liu [20]	Asymmetric	76	0	–	0.9840	0.9780	–	–
Saminathan [21]	SVM	36	24	0.9561	0.9241	0.9650	–	–
Vardasca [22]	kNN	56	0	0.9250	–	–	–	–
Filipe [23]	k-means	122	45	–	–	0.7300	–	0.8100
Eid [24]	kNN	50	0	0.9680	0.9910	0.8830	0.9690	0.9230
Khandakar [25]	AdaBoost	122	45	0.9671	0.9458	0.9671	0.9670	0.9670
Cruz-Vega [19]	DFTNet	110	0	0.9453	0.9375	0.9534	0.9401	0.9457

Several works on the analysis of plantar foot thermograms have been proposed in the past [19–26]. An overview of the different methods, along with performances and datasets used is given in Table 1.

Liu et al. [20] proposed a system for automatic detection of diabetic foot complications using asymmetric analysis. An accompanying color image is used to guide the segmentation as well as non-rigid landmark-based registration to overcome the issue of feet blending in with the surrounding ambient temperature.

Saminathan et al. [21] carried out asymmetric analysis using handcrafted temperature and texture features extracted from the thermograms. A support vector machine (SVM) then used these attributes to identify the region as normal or ulcer.

Vardasca et al. [22] proposed a system based on the k Nearest Neighbours (kNN) classifier. The authors identify the points of the foot with the highest risk of ulceration and compute temperature features at each of these points.

Sudha et al. [26] analyzed the temperature distribution pattern in both healthy and diabetic participants using statistical methods.

Filipe et al. [23] have proposed a binary classifier for distinguishing between thermograms of diabetic and non-diabetic subjects. A temperature index computed using a clustering approach is used for classification between the two groups.

Eid et al. [24] proposed a system that is capable of distinguishing between four grades of diabetic complications. The authors incorporate both histogram and texture features, which are provided to a classifier, and claim that the combined features improve performance. The authors have compared the performance of kNN, SVM and Decision Tree, with kNN having the best results.

Khandakar et al. [25] experimented with several models for binary classification of plantar thermograms into diabetic and non-diabetic subjects. They have also investigated relevant features, feature selection and optimization techniques in order to increase the model performance. The authors compared using a single foot thermogram with a using dual foot thermograms as input and found that the latter performs better. However, this proposed approach reintroduces the previous limitation of not being inclusive of subjects with amputation. The authors report the best performance and smallest inference time by Adaboost classifier coupled with feature selection using random forest.

Cruz-Vega et al. [19] compared the performance of standard machine and deep learning based methods including pre-trained networks against their specifically proposed CNN. Addressing the low number of training samples, the authors propose using patches of the feet rather than the entire image and also enlarging the dataset using data augmentation. The authors performed a multi-level DM grading using One-vs-One classification. The reported results reveal a high discrepancy between the performance of different classes. Moreover, their patch-based approach is likely to disregard important information encoded in a foot’s overall temperature distribution.

Our proposed work consolidates holistic efforts towards practicable DM classification and grading, tackling drawbacks of previous approaches such as the dependence on dual foot imagery and imbalanced classification performance.

## Materials and methods

**Fig. 2:**
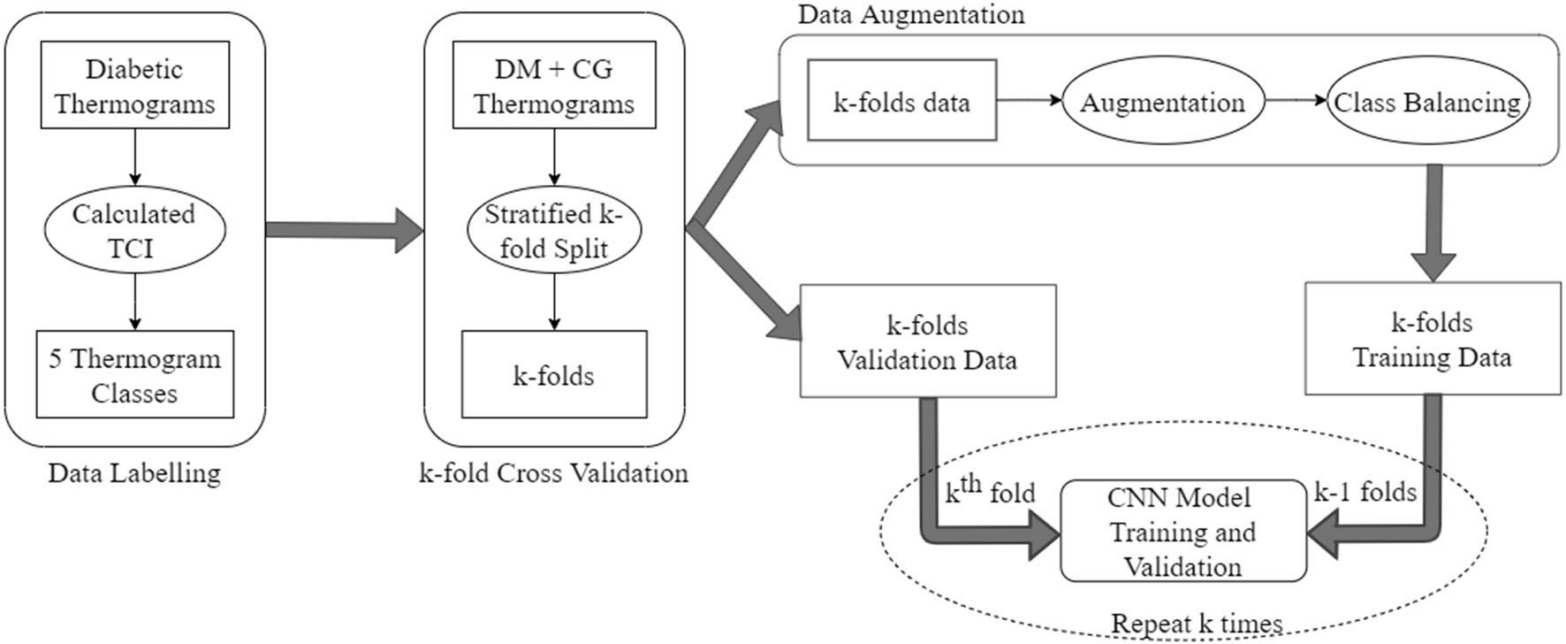
Overview of the methodology depicting the sequence of steps from data preparation to training

In this section, we discuss the dataset used as well as the classification pipeline for our proposed multi-class CNN. Figure 2 depicts the steps involved in the proposed pipeline ranging from data labelling, preparation to model training.

The publicly available dataset used in this study [27] consists of 122 diabetic subjects and 45 control group subjects. The dataset is processed and labeled according to the thermal change index (TCI) introduced in [28]

### Thermal change index (TCI)

**Fig. 3:**
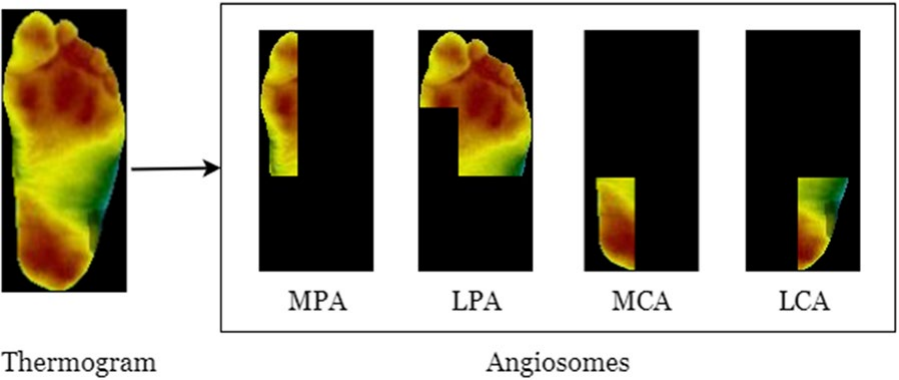
Division of a thermogram into four regions as defined by the angiosomes for computing TCI

TCI is a quantitative index that was proposed by the dataset’s authors in a previous study [28]. This approach proposed that each foot be analyzed independently with the reference butterfly pattern of the control group. As a result, this method is similar to the contralateral comparison or asymmetric analysis, but it is still applicable when there are constraints, such as deformities or amputations. The foot is divided into angiosomes for calculating the TCI. Angiosomes define distinct vascular areas that are supplied by specific arteries. Using the outline defined by the angiosomes, regional plantar temperatures are calculated. Here the foot is divided into four angiosomes, namely medial plantar artery (MPA), lateral plantar artery (LPA), medial calcaneal artery (MCA), and lateral calcaneal artery (LCA). The four angiosomes of the feet are shown in Fig. 3.

The TCI value is the mean temperature difference of corresponding angiosomes between a diabetic subject and the reference values obtained from the control group’s mean temperature per angiosome. For the control group, the mean temperature values were computed and reported as MPA = 25.8 °C, LPA = 25.7 °C, MCA = 26.4 °C, LCA = 26.1 °C. Since each foot is assessed individually using a temperature index derived from angiosome temperature differences, it is not affected by deformities or amputations and does not rely on deviation of symmetry between the feet to determine ulcerations. The computation of TCI for a thermogram is given by the formula below, where ang $\in$ (MPA, LPA, MCA, LCA)

1 $$\begin{aligned} TCI = \frac{\sum _{}^{} |CG_{ang} - DM_{ang} |}{4} \end{aligned}$$

### Thermogram classes

According to the TCI value, the individual thermograms are categorized into one of five classes. Labels are assigned based on the following conditions: Class 1: $TCI \le 2$; Class 2: $2 < TCI \le 3$; Class 3: $3 < TCI \le 4$; Class 4: $4 < TCI \le 5$; Class 5: $TCI >5$.

**Fig. 4:**
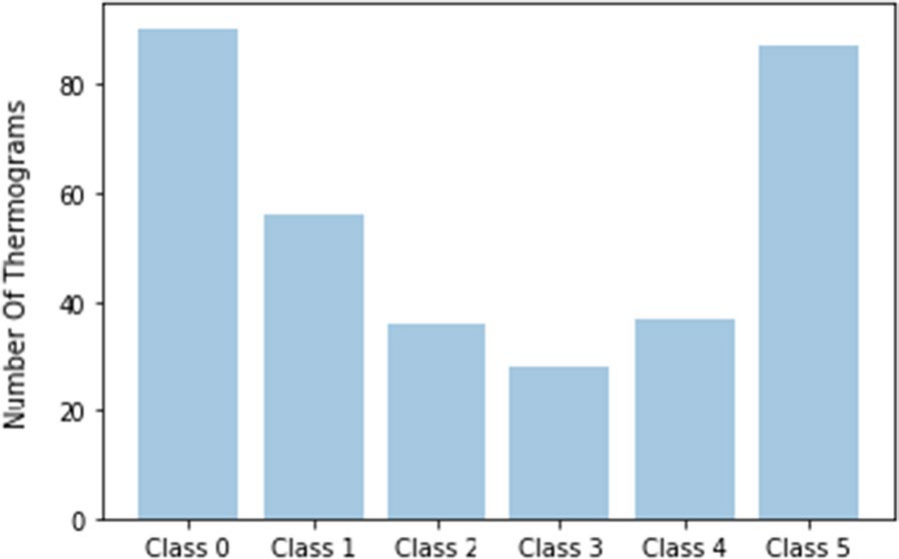
Distribution of thermograms across the six classes

In our study, thermograms from the control group were included as Class 0 in addition to the five classes in diabetic subjects. The class distribution of the raw dataset of individual thermograms is shown in Fig. 4.

### Class balancing

The extremely skewed distribution of thermogram classes poses a high risk of imbalanced classification performance. Imbalanced classification refers to a classification problem in which the number of instances in each class in the training dataset is not evenly distributed. To address the imbalanced dataset, different techniques can be used. Both oversampling and undersampling are not used due to the low number of overall samples present in the dataset, and the risk of overfitting when training from scratch. Advanced techniques for the generation of synthetic data are not trivial in the image space, and are therefore not considered. Instead, we investigate the application of weighted classification as well as data augmentation for balanced classification performance.

Weighted classification: By purposefully adjusting the class weights, a higher importance can be assigned to minority classes. In our experiments, we assign class-weights using the inverse sample frequency for a given target task.

**Fig. 5:**
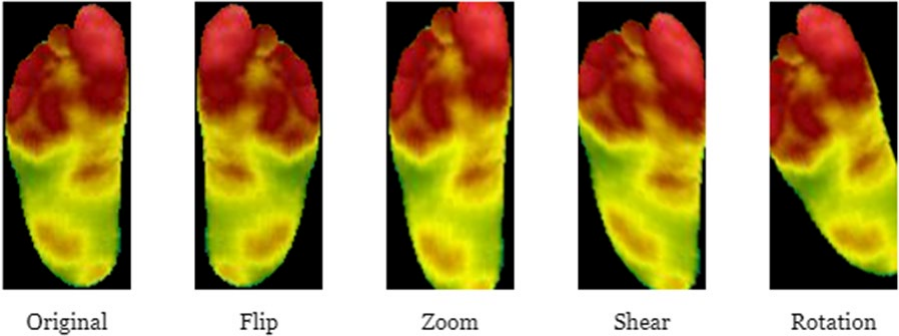
Different augmentation operations applied to a thermogram

Data augmentation: Data augmentation is a simple and effective technique for enlarging a dataset without drastically increasing the risk of overfitting. For each image in the original set, data augmentation generates multiple slightly different versions of images. The utilised augmentation techniques include image rotation, scaling, flipping, and cropping. Figure 5 shows the implemented augmentation operations such as zoom, rotation, flip and shear applied to the thermograms.

Data augmentation can be implemented offline or online. In this study, we first perform offline data augmentation and combine it with the original dataset. In the process, we address the class imbalance by augmenting the minor class more in comparison to the larger classes. This process is applied only on the training data and we preserve the original class distribution in the validation data to make sure the model is assessed in a way that is close to a real world setting. This step of data balancing is critical, as merely augmenting an already-imbalanced data will further drive the imbalance by several magnitudes.

**Fig. 6:**
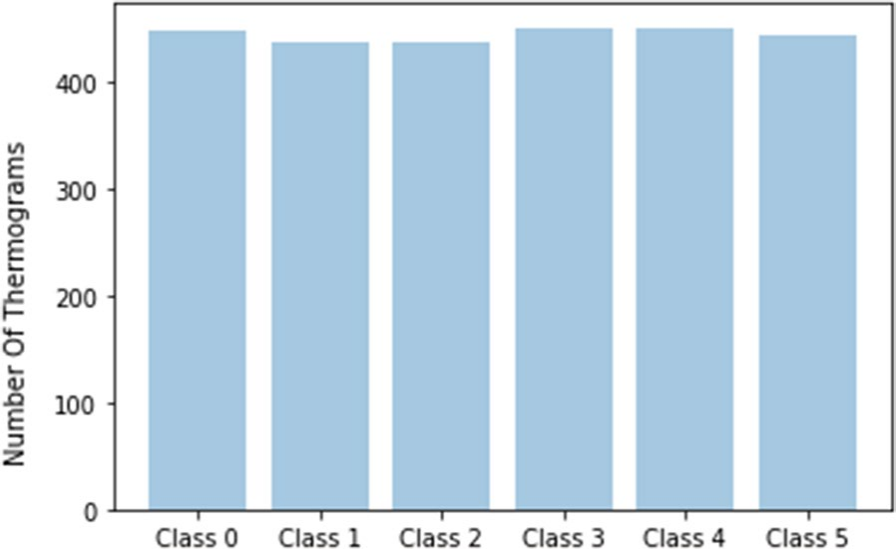
Distribution of thermograms across the six classes after offline augmentation and balancing

Subsequently, we also perform online augmentation as a part of the CNN training process, introducing variations in contrast, brightness, image quality as well as additional affine transforms. Figure 6 depicts the distribution of thermograms across classes following data balancing and augmentation.

We used stratified five fold cross-validation to partition the data. Cross-validation allows for the most optimal comparison with previous works, lacking public data split information, and for the best possible evaluation of model performance under limited data.

### Deep learning model

To alleviate the problems of limited data availability, a common approach is to use transfer learning. Transfer learning is an approach wherein model weights, pre-trained on a related task are *reused* as the starting point for the fine-tuning of the model on a target task. Here we perform transfer learning using a pre-trained AlexNet which takes images in the size of $227 \times 227$.

The feet and correspondingly the dimensions of the plantar thermograms are rectangular in shape. Pre-trained models are rigid in their input requirements, as they need images to be resized into fixed square dimensions. This issue was also discussed by Cruz-Vega et al. [19], where the authors make use of rectangular patches of the foot. Another motivation for using patches instead of the whole thermogram is to increase the size of the data.

**Fig. 7:**
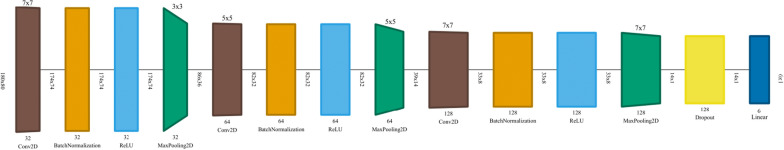
Overview of the proposed CNN architecture describing the layers and their sequence

However, both patch-based transformation and resizing of input images bears the risk of losing patterns and information related to the temperature distribution of the overall thermal image. Therefore, we propose a CNN accepting individual thermograms of size ($180 \times 80$) as input, using a network structure inspired by the work of Cruz et al. [19] as depicted in Fig. 7. The model includes a dropout layer which is randomly enforced during training to avoid overfitting the data.

## Experiments

To accurately measure the model’s performance and expose any biases, the metrics are calculated at the class level. For each class we compute accuracy, specificity, sensitivity, precision, and F-measure.

### Overcoming data imbalance

To assess the effect of the small and skewed dataset, we first train AlexNet with the default parameters on the raw dataset without any external augmentation. Then, we train using weighted classes, where minority classes are given a higher weight, and a misclassification is penalised to a greater degree. Finally, we train on the augmented and balanced data.

The results are reported in Table 2. We observed drastically inconsistent results in different experiments using the raw dataset, with only a marginal improvement in the results using weighted classification. Using a balanced dataset through external augmentation resulted in the best performance across all metrics.

**Table 2:** Addressing small and skewed data through weighted classes and augmentation

Data	Accuracy	Specificity	Sensitivity
Imbalanced data	0.9103	0.9352	0.7283
Weighted classes	0.9154	0.9484	0.7722
Augmented data	0.9335	0.9681	0.8773

### Exploring different input formats

We try improving the results achieved with AlexNet, by using different input formats. The default approach is resizing the images into square images as per the requirements of AlexNet. However, in order to preserve the spatial patterns in the thermograms which would inevitably be distorted by resizing, we use padding to bring the thermogram to the required input size. Finally, we slightly modify the AlexNet architecture to accept rectangular inputs of the size $180 \times 80$.

Table 3 presents the results of training with different input formats. It can be seen that both training with the padded and rectangular input resulted in a better performance across all metrics. This indicates that maintaining thermal patterns in the thermogram is critical for a robust classification. A reason for the lower performance of the rectangular input, is the partial loss of pre-trained weights due to the introduced modifications.

**Table 3:** AlexNet trained with resized, padded and rectangular input images

Input	Accuracy	Specificity	Sensitivity
Resized	0.9335	0.9681	0.8773
Padded	0.9542	0.9710	0.8903
Rectangular	0.9480	0.9686	0.8669

### Holistic classification

We consider two levels of multi-class classification, namely 5-class classification of the diabetic thermograms and a 6-class classification including the non-diabetic thermograms as an additional class. We argue that the holistic consideration of diabetic samples of different grades along with non-diabetic thermograms, adds additional relevant information during the training process, resulting in a more robust classifier. Moreover, this holistic perspective allows for a broader applicability in real-world scenarios.

**Table 4:** Results from 5 vs. 6-class classification

Classes	Accuracy	Specificity	Sensitivity
5-Classes	0.9836	0.9889	0.9583
6-Classes	0.9800	0.9875	0.9583

Table 4 shows that the 6-class classification results in only slightly lower overall performance, although the task’s difficulty is increased. Class-wise metrics of the 6-class classifier for one fold are presented in Table 5. It can be seen that the classification performance is equally distributed between all classes, indicating that the model is not biased towards a single class. When training on the original imbalanced data, a significant model bias towards classes 1 and 5 was observable, with low sensitivity to class 3.

**Table 5:** Class-wise metrics reporting the performance of our proposed holistic model classifying non-diabetic vs. different severity grades of DM

Class	Accuracy	Specificity	Sensitivity	Precision	F-Measure
Class 0	0.9867	0.9968	0.9365	0.9833	0.9593
Class 1	0.9920	0.9936	0.9844	0.9692	0.9767
Class 2	0.9947	0.9936	1.0000	0.9688	0.9841
Class 3	0.9947	0.9968	0.9836	0.9836	0.9836
Class 4	0.9813	0.9840	0.9677	0.9231	0.9449
Class 5	0.9867	0.9968	0.9365	0.9833	0.9593
Average	0.9893	0.9936	0.9681	0.9686	0.9680

**Fig. 8:**
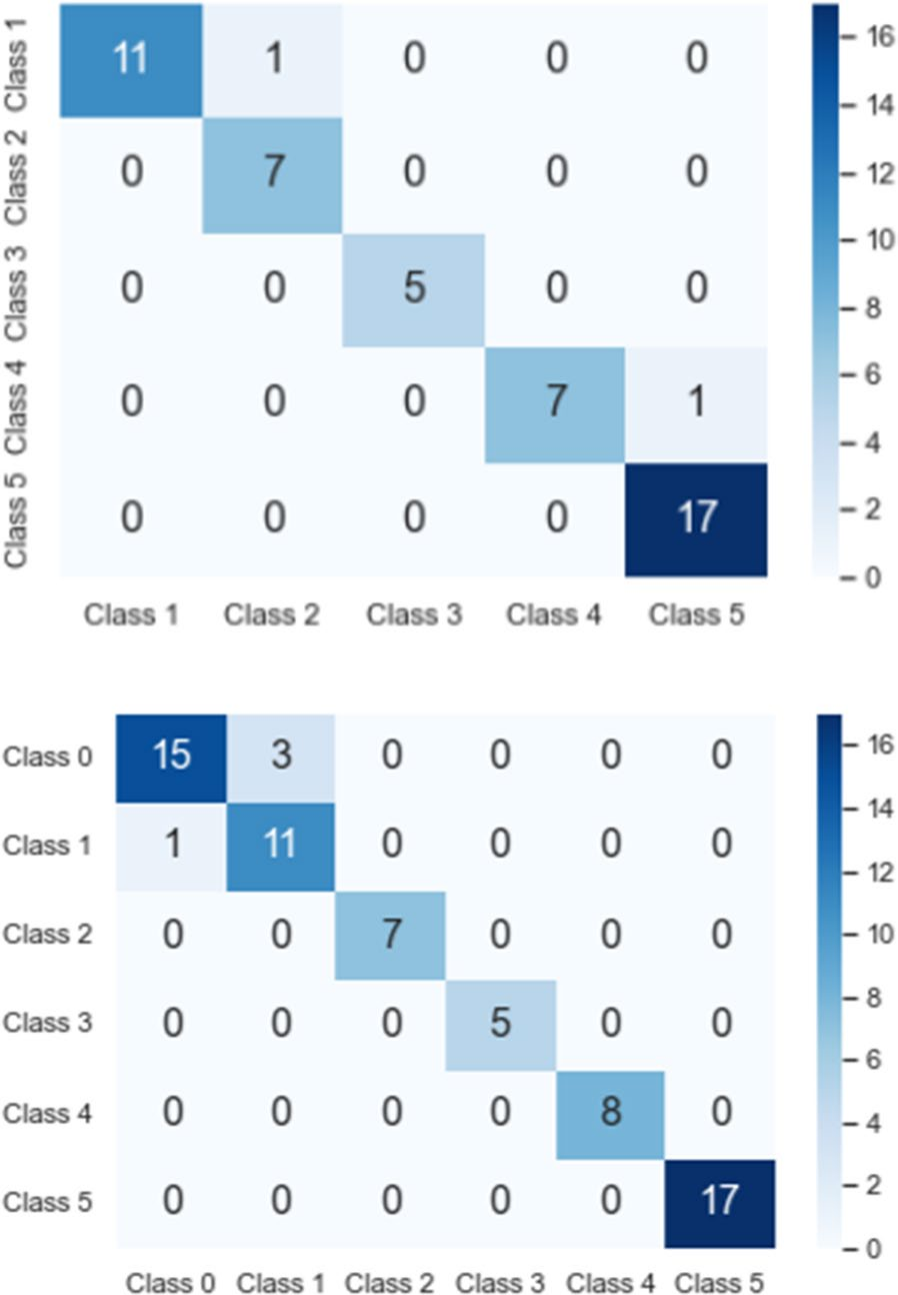
Multi-class confusion matrix from validation split of 5-class vs 6-class classification

From Fig. 8, it can be observed that the performance of the model is largely unaffected even with the inclusion of the control group as the 6th class. The non-diabetic thermograms found in Class 0, are very similar to the thermograms in Classes 1 and 2 in terms of the observed temperature values, but differ in terms of the distribution pattern. As a consequence of using the entire thermogram instead of image patches, the thermal distribution patterns are preserved and the model is able to differentiate between these closely related classes.

Table 6, compares our proposed approach with pre-trained AlexNet as well as all related studies. Alexnet and the proposed CNN were trained with the same data, with input image constraints imposed by the networks taken into account. In Table 6 we listed all approaches that, to the best of our knowledge, proposed relevant works on the publicly available dataset [27] of foot thermograms which has been used in our study. This way, we provide a fair comparison of our method. Due to the lack of other DFU datasets, other comparisons are not possible at this moment. We also make a distinction with respect to the mode of classification. The studies with binary classification differentiate between thermograms of diabetic and non-diabetic subjects. Cruz-Vega et al. [19] have carried out multi-level classification, which involves combinations of one-vs-one binary classifiers covering the five classes. As can be seen from the table, with the best results highlighted in bold, our results outperform previous work on plantar foot thermogram classification on most metrics, although tackling a more difficult 6-class classification task.

**Table 6:** Comparison of our proposed approach with the previous state-of-the-art

Study/model	Classification	Accuracy	Specificity	Sensitivity	Precision	F-Measure
Fillipe [23]	Binary	–	0.7300	–	–	0.8100
Khandakar [25]	Binary	0.9671	0.9458	0.9671	**0**.**9670**	**0**.**9670**
Cruz-Vega [19]	Multi-level	0.9453	0.9375	0.9534	0.9401	0.9457
AlexNet	Multi-class	0.9208	0.9532	0.8440	0.8598	0.8576
Proposed CNN	Multi-class	**0**.**9827**	**0**.**9892**	**0**.**9684**	0.9626	0.9621

## Discussion

A review of the previous literature on plantar foot thermogram analysis revealed a significant imbalance in previously proposed diabetic foot grading systems, caused by highly imbalanced class distributions in public datasets. We explored different methods to overcome the classifier biases induced by imbalanced data and found that a combination of offline and online data augmentation leads to an even distribution of classification metrics. Class-weighted classification potentially failed due to the remaining low diversity of input samples in underrepresented classes.

The experiments in “Exploring different input formats” section clearly indicate the importance of the image integrity for thermogram classification. It is possible that resizing leads to artefacts which corrupt some important pattern in the thermal distribution of the images.

Moreover, the incorporation of non-diabetic subjects turned out to be advantageous for the overall classification performance, leading to a holistic view of plantar foot thermogram detection and grading.

Several studies [29–31] have demonstrated the effectiveness of infrared thermography as a diagnostic tool for the early prediction of ulceration. However, one of the remaining limitations in the widespread use of thermography for the detection and grading of diabetic subjects is the lack of standardized thermal imaging. The range used to indicate the temperature value differs across acquisition systems. This lack of a standard methodology hinders the generalizability and practical implementation of such decision support systems. Another challenge in assessing the generalizability of proposed algorithms is the dearth of publicly available datasets for evaluation. To the best of our knowledge, the dataset utilized in this study is the only one that is publicly available, to this date.

## Conclusion

Amputations resulting from diabetic foot ulcerations can be prevented if the ulcerations are diagnosed early on and corrective methods such as pressure offloading, special orthopedic shoes, or a modification in gait are implemented.

This study is the first to present a holistic multi-class classification of thermal plantar foot images for the prediction and grading of diabetic subjects. In contrast to previous work, considering only binary classification or severity grading, we organized thermograms into six classes, depending on the TCI value, offering a more practical and holistic view on the problem. Publicly available data is scarce and often imbalanced, leading to a bias towards overrepresented classes and low sensitivity towards underrepresented classes. We addressed this issue by investigating different methods preventing imbalanced classification, explored the influence of different data input formats for thermograms, and compared transfer learning with common CNNs to the application of custom architectures.

Our results indicate that a mixture of offline and online data augmentation is best suited for detection and grading of diabetic subjects. We also found that maintaining the original aspect ratio of thermogram images is extremely important. To the best of our knowledge, this study sets a new state-of-the-art for the detection and grading of plantar foot thermograms, achieving a mean accuracy of 0.9827, mean sensitivity of 0.9684 and mean specificity of 0.9892.

As future work, we would like to evaluate previous and our proposed methods for the prediction of exact ulceration points from the thermogram data. Another promising direction is the inclusion of clinical data with thermogram imagery to predict the likelihood of amputations.
